# Stress Biomarkers in Medical Students Participating in a Mind Body Medicine Skills Program

**DOI:** 10.1093/ecam/neq039

**Published:** 2011-06-08

**Authors:** Brian W. MacLaughlin, Dan Wang, Anne-Michelle Noone, Nan Liu, Nancy Harazduk, Michael Lumpkin, Aviad Haramati, Pamela Saunders, MaryAnn Dutton, Hakima Amri

**Affiliations:** ^1^Department of Physiology and Biophysics, Georgetown University Medical Center, 3900 Reservoir Road, Washington, DC 20007, USA; ^2^Department of Biostatistics, Bioinformatics, and Biomathematics, Georgetown University Medical Center, 3900 Reservoir Road, Washington, DC 20007, USA; ^3^Department of Mathematics, Georgetown University Medical Center, 3900 Reservoir Road, Washington, DC 20007, USA; ^4^Department of Neurology, Georgetown University Medical Center, 3900 Reservoir Road, Washington, DC 20007, USA; ^5^Department of Psychiatry, Georgetown University Medical Center, 3900 Reservoir Road, Washington, DC 20007, USA

## Abstract

Georgetown University School of Medicine offers an elective Mind-Body Medicine Skills (MBMS) course to medical students to promote self-care and self-awareness. Participating medical students reported better management of academic stress and well-being than non-participants. In this study, we sought to assess the stress-reducing effects of MBMS by measuring physiological changes in first-year medical students. Saliva samples were collected before (January, time 1 (T1)-pre-intervention) and upon completion of the course (May, time 2 (T2p)-post-intervention), as well as from non-participating medical students (May, time 2 (T2c)-control). The T2p and T2c collections coincided with the period of final examinations. Cortisol, dehydroepiandrosterone-sulfate (DHEA-S), testosterone and secretory immunoglobulin A (sIgA) were measured. The mean morning salivary cortisol at T2p was 97% of the mean at baseline T1 which was significantly lower than for T2c (2.4) (95% confidence interval (CI) 0.57–1.60, *P* =  .001); DHEA-S showed similar pattern as cortisol where the T2p levels were significantly lower than T2c (*P* <  .001) in both morning and evening collections. Testosterone ratio at T2p (0.85) was also lower than T2c (1.6) (95% CI 0.53–1.3, *P* =  .01). sIgA levels were not statistically different. On direct comparison, the T2c and T2p means were significantly different for all cortisol, DHEA-S and testosterone values. Participants maintained their hormonal balance within the normal range throughout the academic semester while the control group showed significantly increased levels, probably exacerbated by the end of the semester exam stress. To our knowledge, this is the first study to assess the physiologic benefits of a MBMS program in medical students.

## 1. Introduction

High stress, common to the medical profession, can lead to physical exhaustion and mental fatigue among doctors. A study of New Zealand physicians found that 28% reported high levels of emotional exhaustion and depersonalization or low personal accomplishment, which are both components of burnout. Of special concern is the negative correlation between stress in the medical profession and the quality of patient care. In an anonymous questionnaire, one-third of 225 responding doctors reported lower standards of patient care due to stress, with 10.5% resulting in serious mistakes [1]. It is well documented that stress in doctors starts prior to the commencement of their professional careers; high levels of academic stress are reported amongst undergraduate medical and pre-medical students. A review by Dyrbe et al. highlighted the many stressors unique to medical school, which range from the high volume workload to ethical conflicts, and exposure to human suffering and death. These stressors can lead to impaired academic performance, cynicism, academic dishonesty, substance abuse and even suicide [2].

Considering the impending future doctor shortage [3], stress in the medical profession is only likely to increase. Strains on the healthcare system impair physicians' ability to manage their stress and negatively affect their performance, thus making the promotion of personal well being of the utmost importance. The practitioner's well-being could be enhanced by promoting self-awareness and self-care that could be achieved by practicing Mind-Body Medicine (MBM). MBM takes into account the connectedness between the mind and body, and its effect on overall health. It embraces several well-defined strategies such as relaxation, meditation, yoga, biofeedback, imagery, autogenic training, tai chi, qi gong, hypnosis and spirituality. A study by Nakao et al. at a Mind-Body clinic in Massachusetts found significant decreases in medical symptoms, as assessed by the medical symptom checklist including the 12 major symptoms, and improvements in stress perception among 911 adult participants referred to the clinic [4]. A stress management intervention program for 15-16-year-old students in the UK improved academic performance and general mental health [5]. Finkelstein et al. [6] studied anxiety and stress parameters among medical students using self-report instruments examining stress, anxiety, mood states and depression; they found the initially high levels of stress and anxiety among a group of students enrolled in a MBM course decreased to the level of a peer control group after course completion. A study by Shapiro et al. of pre-medical and medical students used questionnaires to measure empathy, psychological distress, depression, state and trait anxiety and spirituality before and after participation in a 7-week mindfulness-based intervention program. The study found reductions in self-reported scores of depression, state and trait anxiety, and increases in scores of empathy and spirituality compared to a randomized wait-list control group of students equally interested in participation in the program [7].

Georgetown University School of Medicine (GUSOM) recognizes physician well-being as an important factor in administering proper medical care. In order to best prepare its future physicians, GUSOM has implemented an elective Mind-Body Medicine Skills (MBMS) program for its medical students throughout the undergraduate years. The MBMS program seeks to teach medical students methods of stress management for use during their medical studies and in their careers. By using questionnaires that included six open-ended questions, a qualitative study conducted at GUSOM found that MBMS promotes students' ability to achieve better stress management, self-awareness and self-care.

**Figure 1:**
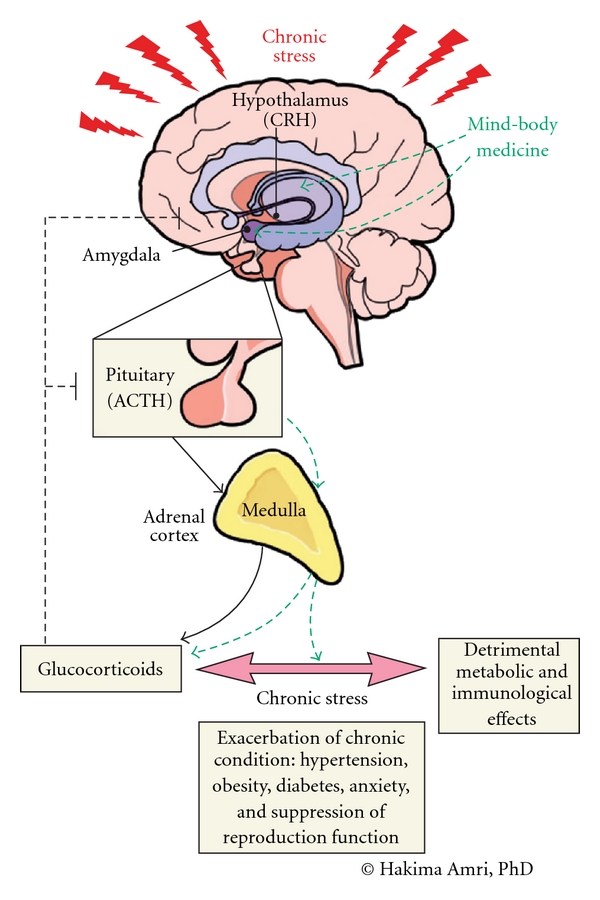
Schematic representation of the regulation of the HPA axis under chronic stress. CRH and arginine vasopressin (AVP) synthesized by the paraventricular nucleus and released to the hypophyseal portal system stimulate adrenocorticotropin hormone (ACTH) secretion by the anterior pituitary. ACTH triggers glucocorticoids release from the adrenal cortex. In an acute stress response, glucocorticoids regulate CRH and ACTH release in a negative feedback loop. However, in chronic stress, sustained glucocorticoids synthesis becomes detrimental to metabolic, endocrine, and immunologic processes leading to pathological states. MBM plays a role in maintaining stress hormone levels within their normal range. MBM may also affect the release of CRH and ACTH by helping to quiet the mind. Solid arrows: positive regulation, dotted lines: negative feedback, and dotted arrows: normalizing effects.

In addition to having an impact on psychological health, physical and mental stressors, including academic stress [8, 9], the practice of MBMS has been shown to cause shifts in the neuro-endocrine regulatory systems governed by the hypothalamic-pituitary-adrenal (HPA) axis and the sympathetic nervous system (SNS) [10]. The HPA axis plays a major role in conveying the central stress response to the peripheral body systems to maintain a homeostatic balance. The diverse expression of corticotrophin-releasing hormone (CRH) receptors attests to CRH's pivotal role in the modulation of stress at different levels such as appetite control, immune response and cardiovascular function. Glucocorticoids secreted by the adrenal cortex exhibit various phenotypic effects through the wide distribution of their intracellular receptors [11] that regulate a number of genes involved in different metabolic functions [12, 13]. In acute stress, increased glucocorticoids mobilize all responsible body systems to respond to the stressor in the best possible manner [14, 15]. However, if the stress is chronic, maintained high glucocorticoid levels cause marked shift in energy stores towards the center of the body [16], hypertension [17], cardiovascular problems [18], metabolic syndrome [19] and suppression of the reproductive function [20]. In addition, sustained high glucocorticoid levels maintain the brain in an alert and anxious state that could lead to inappropriate learning, memory processing and decision making [21, 22]. Cortisol is the principal glucocorticoid hormone involved in the stress response (Figure 1). 

It is well documented that the response of the HPA axis and its stress mediators occurs according to a non-linear network, which makes the design of a pharmacological compound that acts on the different bodily systems with minimal side effects a great challenge [23]. Therefore, employing strategies such as MBM that act on higher cognitive brain areas and peripheral processes without exhibiting side effects, could be the therapeutic strategy of choice. To date, very limited information is available on the beneficial effects of MBM on the HPA axis and its stress mediators in a healthy population exposed to chronic academic stress.

In the current pilot study, we sought to assess the GUSOM MBMS program in regard to quantifiable physiologic and immunologic stress markers. Salivary cortisol, DHEA-S, testosterone and secretory immunoglobulin A (sIgA) were assayed in first-year medical students before and after MBMS program and compared to control medical students not participating in the program. To our knowledge, no other studies have reported on the physiological impact of the MBMS program.

## 2. Methods

### 2.1. Medical Student Population

All study participants were first-year medical students enrolled during the spring semesters of the academic years 2003–04 and 2004–05. Students were invited to participate in an elective MBMS course that met once a week for 2 h over a period of 11 weeks. Students participating in the MBMS course were asked on a voluntary basis to provide two saliva samples (morning and evening) both prior to starting the MBMS course (January, Time1 (T1)-pre-intervention), and at completion of the course, which occurred near the end of the spring semester (May, Time 2 (T2p)-post-intervention). The T2 collection coincided with a final examination period for the spring semester. The control group consisted of first-year medical students who were not enrolled in the MBMS course, and all samples were collected at a concurrent time (May, Time 2-control (T2c)). The study was approved by the Georgetown University Institutional Review Board and informed consent was obtained from all students submitting saliva samples.

### 2.2. Mind-Body Techniques

The MBMS program at GUSOM is an adaptation and modification of the Mind-Body-Spirit program initially developed at the Center of Mind-Body Medicine [24] and includes elements of the Mindfulness-Based Stress Reduction (MBSR) program [25, 26]. The MBMS program consists of 11 weekly group sessions that are skill-based and experiential, each accompanied by a group discussion. Exercises include: autogenic training (the use of verbal commands to bring about a relaxation response); biofeedback (use of measuring devices such as the thermistor to provide visual feedback of physiologic changes); imagery (use of sensory imagination to develop symbolic representation of healing the mind, body and spirit); journal writing (enhance self-awareness and self-reflection); and meditation (using focus and self-observation to achieve greater awareness of the present moment). Typical sessions begin and end with guided meditation, discussion of learned techniques and introduction of new skills. Students are expected to continue journal writing and meditation practices independently throughout the week. Adherence to the intervention protocol was above 98%; basically participating students did not miss any of the 11 sessions.

### 2.3. Saliva Collection

Students who volunteered to give saliva samples were provided with two 15 mL conical graduated polystyrene tubes (Fisher Scientific, PA). They were then instructed to collect two saliva samples of about 6–8 mL each, one in the morning between 7 and 8 AM, and one in the evening between 10 PM and 12 AM. Students were directed not to eat, drink, or brush teeth for an hour prior to collection, to avoid contamination of saliva with food debris or blood, to rinse mouth with water prior to collecting sample, and to freeze saliva samples before submitting to our laboratory. All samples were kept frozen in our laboratory until they were analyzed.

### 2.4. Hormone Testing

All saliva samples were thawed to room temperature and centrifuged at 1200 × g for 10 min before testing. Salivary cortisol, testosterone and DHEA-S were measured by enzyme linked immunosorbent assay (ELISA) according the manufacturer's instructions (DiaMetra). Detection ranges: (i) cortisol: 0.5–100 ng mL^−1^, with reference values for the AM 3–10 ng mL^−1^ and PM 0.6–2.5 ng mL^−1^; (ii) testosterone: 5–1000 pg mL^−1^, with reference values for normal women 10–55 pg mL^−1^ and for normal men 50–210 pg mL^−1^; (iii) DHEA-S: 0.2–12 ng mL^−1^, with reference values for women 0.2–2.5 ng mL^−1^ and men 0.2–2.7 ng mL^−1^; and (iv) sIgA was measured with ELISA from Salimetrics LLC with a detection range 2.5–600 *μ*g mL^−1^.

### 2.5. Statistical Analysis

Hormone levels were expected to increase from January to May in the general medical student population due to the academic load and exams [27]; so the average change in hormone levels was estimated as the change in January using hormone levels from all participants at T1 to the hormone levels of the control group in May T2c. Since participants in the MBMS course were expected to have their hormonal levels within the normal range throughout the semester, they were not included in the estimation of the overall change. The effect of the MBMS course on hormone levels was first assessed by comparing the average semester change among those enrolled in the course to the overall mean change. Comparisons were performed using paired *t-*tests in which the null hypothesis was the estimated overall mean hormonal change during the semester. In addition, *t-*tests were used to compare the mean hormone levels in May (T2c) among those in the control group to those enrolled in the MBMS course (T2p). All hormone levels were transformed using the natural logarithm in order to meet normality assumptions for analysis. The results were back-transformed and presented on the original scale. Note that differences on the logarithmic scale become ratios when back-transformed to the original scale. All statistical tests were two-sided and performed using SAS software, Version 9.1 (SAS Institute, Inc., Cary, NC).

## 3. Results

### 3.1. MBMS Course Participants

**Figure 2:**
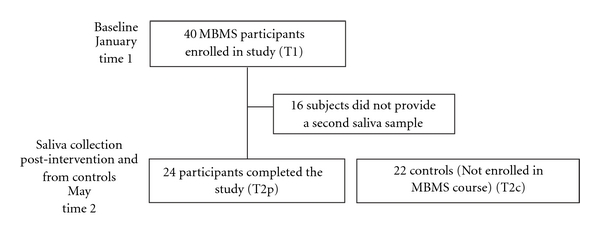
Flowchart summarizing the collection times and participation in the study.

Since GUSOM started offering the MBMS elective course in 2002, over 30% of first-year medical students enroll every year. During the two academic year period of the study, 40 of all the enrolled students (including 32 females) volunteered to participate and provide a saliva sample at T1. Of these students, 24 (including 20 females) provided a second set of saliva samples at T2p. In addition, salivary samples of 22 controls not participating in the MBMS course, including 10 females, were collected in May at T2c (Figure 2). Levels of cortisol, testosterone, DHEA-S and sIgA were determined and comparisons among intervention and no intervention groups were performed. 

### 3.2. Cortisol

**Figure 3:**
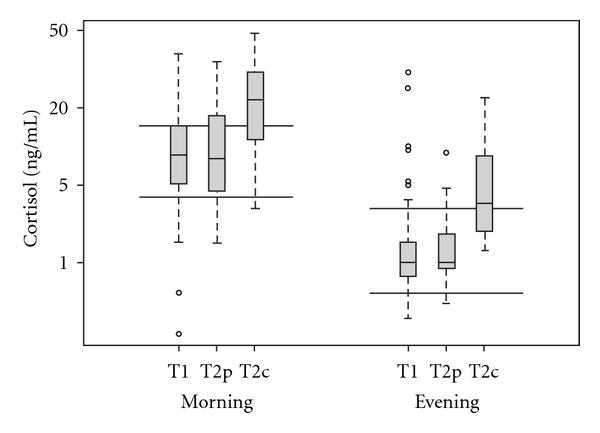
Log-transformed salivary cortisol levels measured in morning and evening samples with axes labeled on the original scale. Parallel bars represent the range of normal cortisol levels. The lower edge of the box represents the first quartile, the mid-line is the median, and the upper edge is the third quartile.

The mean morning cortisol in the control group at T2c was 2.4 times higher than the January baseline at T1. The change in morning cortisol among the MBMS group at T2p was statistically significantly less than the increase observed in the control group. The control group was 2.4 times higher at T2 whereas the MBMS group was only 0.97 times higher at T2. Specifically, at T2p the mean morning cortisol for students who received MBMS was 97% (95% confidence interval (CI) 57–160%) of the mean morning cortisol at T1 (*P* =  .001, Table 1). In addition, the mean morning cortisol values of the MBMS group at T2p was significantly lower than the mean of controls at T2c (*P* =   .002). Specifically, the control group had a mean morning cortisol of 14 ng mL^−1^ (95% CI 10–19), which was significantly elevated compared to the mean for the MBMS group at T2p of 6.6 ng mL^−1^ (95% CI 4.6–9.5) (Table 2). The mean for the control group was out of the normal morning range of 3–10 ng mL^−1^ for cortisol, while both groups at T1 and T2p, respectively, had means within the normal range. Similarly, the mean evening cortisol at T2p was 76% (95% CI, 42–140%) of the mean evening cortisol at T1, which contrasted with the 240% increase among the controls at T2c (*P* =  .001). Comparison of the mean evening cortisol levels showed a similar trend as the morning, with levels of the control group significantly higher than participants in MBMS course. The control mean evening cortisol level was also out of the normal range at 0.6–2.5 ng mL^−1^, while T1 and T2p mean levels were within the normal range (Figure 3). No difference in cortisol levels was found between male and female students. In addition, no difference was noted at baseline between the students who provided the second saliva samples and those who did not. 

### 3.3. DHEA-S

**Figure 4:**
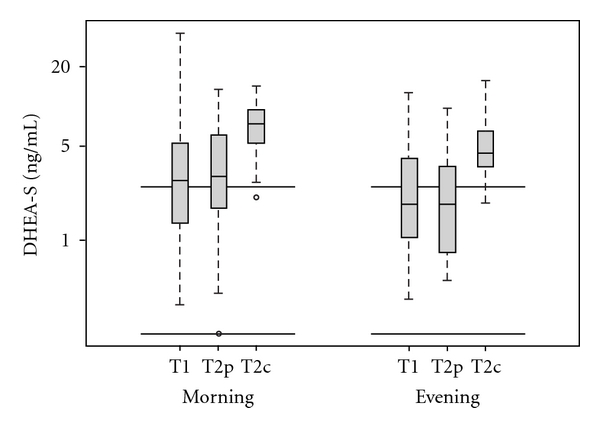
Log-transformed salivary DHEA-S levels measured in morning and evening samples with axes labeled on the original scale. The parallel bars represent the range of normal DHEA-S levels. The lower edge of the box represents the first quartile, the mid-line is the median, and the upper edge is the third quartile.

Similar to the cortisol profile, the mean morning levels for DHEA-S in the control group at T2c were higher than the levels at the baseline T1. Compared with T1, mean DHEA-S level was 2.5 times higher among the control group at T2c, while levels among the MBMS group at T2p did not change (ratio = 1.0, (95% CI 0.74–1.4), *P* <  .001) (Table 1). Evening DHEA-S salivary amounts were also, on average, 2.4 times higher at T2c while T2p values remained within the physiological range (ratio = 0.81, (95% CI 0.56–1.2), *P* <  .001). In addition, comparison of mean DHEA-S levels in May between T2p and T2c were also significantly different; both morning and evening control values were higher on average than MBMS participants (morning: 6.6 ng mL^−1^ (95% CI 5.3–8.1) and 2.9 ng mL^−1^ (95% CI 1.9–4.4), *P* =  .001; and evening: 4.8 ng mL^−1^ (95% CI 3.8–6.0) and 1.9 ng mL^−1^ (95% CI 1.3–2.9 ng mL^−1^), *P* <  .001, resp.) (Table 2). DHEA-S levels were above the higher limit of the physiologic range in all groups, probably due the young age of the subjects (Figure 4). 

### 3.4. Testosterone

**Figure 5:**
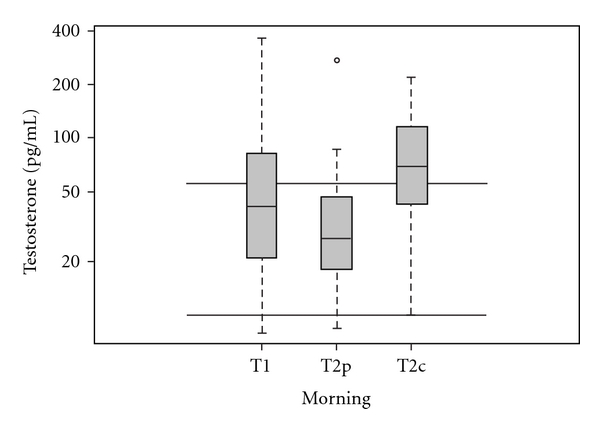
Log-transformed salivary testosterone levels measured in morning samples among female subjects with axes labeled on the original scale. The parallel bars represent the range of physiological testosterone levels. The lower edge of the box represents the first quartile, the mid-line is the median, and the upper edge is the third quartile.

Only testosterone values for the female students, in both groups, were calculated and presented here due to the limited number of available male samples. The average morning testosterone values at T2c was 1.6 times higher than baseline T1 whereas the mean level at T2p was only 85% (95% CI 53–130%) of the mean morning testosterone at T1, *P* =  .01 (Table 1). The mean evening values for the MBMS group T2p was about equal to the values at T1, however this was not significantly different from the estimated change among T2c (*P*  =  .07); nevertheless levels at T2c remained elevated (1.6), while those at T2p showed no change (ratio = 1.0 (95% CI 0.60–1.7)). When comparing mean hormone amounts between T2c and T2p, we observed significant differences for both morning and evening testosterone values. Morning MBMS at T2p (30 pg mL^−1^ (95% CI 21–44)) was 53% lower than control at T2c (64 pg mL^−1^ (95% CI 34–121), *P* = .03). The evening testosterone amounts at T2p ((28 pg mL^−1^ (95% CI 20–41)) were also 52% lower than levels at T2c (54 pg mL^−1^ (95% CI 42–70), *P* =  .02) (Table 2). Mean morning testosterone at T2c tested out of the normal female range of 10–55 pg mL^−1^ (Figure 5). 

### 3.5. sIgA

Neither the morning nor evening mean change in sIgA levels of MBMS to baseline at T1 were significantly different than the mean change observed in the control group. Similarly direct comparison of the mean hormone levels in May between control and MBMS were not significantly different (*P* =  .05) (Tables 1 and 2).

## 4. Discussion

Analysis for cortisol levels for both morning and evening collections at T2p from students in the MBMS course did not differ significantly from baseline values at T1, and hormone levels from both of these groups were in the normal range. In addition, these students seemed to be protected from the significant increase in cortisol levels that was found in the control group at T2c during the high stress period of final examinations. Increased cortisol in medical students in relation to examination stress is a finding noted previously by other authors [14]. In a recent study investigating salivary cortisol as an HPA activity marker and salivary alpha-amylase as the SNS marker, undergraduate students have been monitored before and after taking an oral exam. A strong anticipatory increase in both cortisol and salivary alpha-amylase was detected indicating academic stressors indeed modulate the neuroendocrine system [28]. Using second-year medical students, van Dulmen et al. assessed the impact of simulated bad news consultations on psycho-physiological stress. Among the physiological parameters measures, salivary cortisol was measured on the assessment day and compared to the collections from a quiet control day. Cortisol levels remained elevated suggesting a sharp anticipatory stress response [29]. Furthermore, undergraduate students enrolled in the General and Comparative Endocrinology course were asked to participate in a study assessing the stress response following a presentation competition and fasting. Salivary cortisol concentrations were elevated in response to all three stressors when compared to their matched basal conditions [30]. Also, focusing on the examination stress as a trigger of the stress response, Weekes et al. [27] administered psychological inventories to and collected salivary samples from undergraduate students. They found that examination stress increased cortisol levels and elevated psychological measures. All these findings are in agreement with our results that cortisol levels increase during examination time. Thus, our control group not participating in the MBMS course exhibited high levels of cortisol coinciding with the examination period of the spring semester compared to the students who learned MBM skills that helped them manage their stress. These findings are in agreement with our previously reported qualitative analysis that showed better stress management and coping with the use of MBMS [31].

DHEA-S and cortisol showed a parallel diurnal secretory pattern. Thus, morning DHEA-S levels in the MBMS group participants were significantly lower by 56% and the evening values by 60% than in the control group not participating in the MBMS program. However, DHEA-S secretion is also age dependent, with levels peaking between the ages of 20 and 30 years and decreasing with age [32]. This age range encompasses the medical students in our study, and most likely explains the consistent readings above the available normal range in all groups. In another study, male students have been subjected to psychosocial stress where they have been asked to give a speech and perform arithmetic calculations in front of two audiences. In addition to measurements of autonomic nervous system parameters (blood pressure and heart rate) and psychometrics, saliva samples were collected for cortisol and DHEA quantification. Izawa et al. found that this stressor increased DHEA level by 60% immediately after completion of the tasks. They also showed that DHEA and cortisol responses were correlated while no correlation was observed to the cardiovascular response [33]. Mommersteeg et al. found increased DHEA-S related to burnout from chronic work stress in subjects who were on sick leave with a neurasthenia diagnosis [34]. In another study that examined the effects of stress on HPA axis hormones during military survival training, the authors reported that both cortisol and DHEA-S increased significantly during the stressful captivity phase of the training compared to a free-living environment [35]. Findings of all the above-cited studies are congruent with our results that DHEA-S increased in subjects exposed to a stressful environment and that it is correlated to cortisol levels. To our knowledge, this is the first study to show the impact of MBM on DHEA-S levels in a challenging academic setting.

We found significantly increased morning testosterone levels among the control group of females over female MBMS participants when comparing hormone ratios to the baseline levels. Testosterone levels were 113% significantly higher among the control group when directly compared to T2p MBMS group. Our results may add to the sparse evidence linking stress to increased testosterone in women. Asberg et al. screened 195 women on long-term sick leave complaining of burnout syndrome, exhaustion and other stress related disorders for 17 biological mediators encompassing hormones, cytokines and growth factors. Plasma testosterone was found elevated in the sick leave group [36].

Daily production of immunoglobulin A is higher than all other antibodies combined, and sIgA, the major antibody in defense against pathogens in mucosal surfaces, seems to be affected by stressful stimuli [37]. A meta-analysis study reported on the correlations between stress defined as negative life events and immune parameters including sIgA, serum immunoglobulins, and cells of the immune system. In particular, stress was associated with decreased sIgA [38]. In our study, no significant differences were noted between the control and MBMS groups. sIgA levels were shown to be slightly reduced during a prolonged period of a major examination among medical students, and were significantly reduced for weeks following the examination period [39].

The robustness of the outcomes of this pilot study could have been strengthened by a larger number of subjects in each comparison group and including a balanced number of both genders as well as controlling for possible covariates such as blood pressure, body mass index, insulin and glucose levels. In addition, measuring hormone levels in May, in the same control group from baseline, would have provided a stronger study design, and allowed for a direct comparison in change of hormone levels between the intervention and control groups. Future studies may benefit from multiple daily samplings as well as at mid-point saliva collection during the intervention. Future studies could also benefit from a random assignment to study groups even within the self-selected students that enroll in MBMS program. The latter could benefit from a wait-listed group.

In conclusion, GUSOM has taken the initiative to make management of one's own well being a focus for its medical students. Teaching future physicians a set of skills to cope with the stress of their profession will likely aid both their future career success and personal well-being. Academic stress in medical schools not only has an immediate impact on academic performance, but can also lead to cynicism in the form of decreased empathy and humanitarianism [40]. In this study, the recorded physiological parameters of stress correlate well with perceived psychological benefits of MBM, both previously reported from the MBMS course at Georgetown University as well as other institutions [6, 31]. It is important to keep in mind that the participating students of this study, while under considerable academic stress, were generally young and healthy and did not exhibit any health problems during the study. Even in this population, MBM techniques result in quantifiable improvements in physiological markers of stress. Those with chronic conditions, either due to or exaggerated by stress, may stand to derive an even greater benefit.

## Figures and Tables

**Table 1:** Ratio of mean May hormone levels (T2c) relative to January baseline (T1) compared with the overall ratio.

	Time of day	*N*	Overall ratio of mean T2 hormone levels to T1	Ratio (95% CI) of mean T2c hormone levels to T1	*P-*value*
Cortisol	Morning	24	2.4	0.97 (0.57, 1.6)	.001
	Evening	22	2.4	0.76 (0.42, 1.4)	.001
DHEA-S	Morning	24	2.5	1.0 (0.74, 1.4)	<.001
	Evening	22	2.4	0.81 (0.56, 1.2)	<.001
Testosterone	Morning	20	1.6	0.85 (0.53, 1.3)	.01
	Evening	18	1.6	1.0 (0.60, 1.7)	.07
sIgA	Morning	24	1.4	1.7 (1.2, 2.4)	.43
	Evening	22	1.2	1.3 (0.92, 1.9)	.65

*P-values were computed using paired *t-*tests that compared the ratio among the intervention group to the estimated overall ratio.

**Table 2:** Mean hormone levels in May for the control and MBMS group.

	Time of day	Controls			MBMS			*P*-value
		*N*	Mean	95% CI	*N*	Mean	95% CI	
Cortisol	Morning	22	14	(10, 19)	24	6.6	(4.6, 9.5)	.002
	Evening	22	3.2	(2.3, 4.4)	22	1.2	(1.0, 1.6)	<.001
DHEA-S	Morning	22	6.6	(5.3, 8.1)	24	2.9	(1.9, 4.4)	.001
	Evening	22	4.8	(3.8, 6.0)	22	1.9	(1.3, 2.9)	<.001
Testosterone	Morning	10	64	(34, 121)	20	30	(21, 44)	.03
	Evening	10	54	(42, 70)	18	28	(20, 41)	.02
IgA	Morning	22	189	(137, 260)	24	161	(100, 261)	.58
	Evening	22	100	(74, 135)	22	106	(76, 147)	.79
